# Prevalence and correlates of teenage pregnancy among in-school teenagers during the COVID-19 pandemic in Hoima district western Uganda–A cross sectional community-based study

**DOI:** 10.1371/journal.pone.0278772

**Published:** 2022-12-16

**Authors:** Marvin Musinguzi, Edward Kumakech, Anne Grace Auma, Ruth Anne Akello, Eustes Kigongo, Raymond Tumwesigye, Bosco Opio, Amir Kabunga, Bernard Omech

**Affiliations:** 1 Department of Public Health, Lira University, Lira, Uganda; 2 Department of Nursing and Midwifery, Lira University, Lira, Uganda; 3 Department of Psychiatry, Lira University, Lira, Uganda; University of Nairobi, KENYA

## Abstract

**Background:**

The COVID-19 pandemic related restrictions and lockdown measures had compromised the routine delivery and access of sexual and reproductive health and rights services to the population including the teenage girls. However, the teenage pregnancy rates during COVID-19 pandemic period were poorly documented. This study aimed at determining the prevalence and the factors associated with teenage pregnancy among in-school teenage girls during the COVID-19 pandemic period in Hoima District Uganda.

**Methods:**

This was a descriptive cross-sectional study that employed quantitative research methods. A total of 314 in-school teenage girls aged 13–19 years were selected using a multi-stage sampling techniques. Interviewer-administered questionnaires were used to collect the data from the participant’s homes during the period December 2021-January 2022. Data analysis was done using univariate, bi-variate, and multivariate.

**Results:**

The prevalence of teenage pregnancy among the in-school teenage girls in Hoima district Uganda was 30.6% [96/314]. Higher teenage pregnancy rates were prevalent among the unmarried teenage girls [aOR: 9.6; 95%CI: 4.64–19.87; p = 0.000], teenage girls studying from boarding schools [aOR 2.83, 95%CI 1.36–5.86, p = 0.005], contraceptive non-users [aOR: 2.54; 95%CI: 1.12–5.4; p = 0.015] and teenage girls involved in sex trade [aOR 3.16, 95%CI 1.5–6.7, p = 0.003]. The factors associated with the reduced likelihood for teenage pregnancy included being an adult teenage girl aged 18–19 years [aOR: 0.15; 95%CI: 0.07–0.32; p = 0.000] and not receiving sex education during the period [aOR 0.36, 95%CI 0.13–0.62, p = 0.024].

**Conclusion:**

The results indicated that 3 out of 10 in-school teenage girls from Hoima district Uganda got pregnant during the COVID-19 pandemic period of 2021. Teenage pregnancy was prevalent among teenage girls who don’t use modern contraceptive methods and those involved in sex trade. Teenage pregnancy was however, less prevalent among adult teenage girls aged 18–19 years. The findings point to the need for health stakeholders to innovate creative policies, contingency plans and programmes aimed at delaying age for sexual activities, increasing contraceptive use and minimizing pregnancy risk from sex trade among in-school teenage girls during COVID-19 pandemics.

## Introduction

Teenage pregnancy is a public health concern with 15% of teenage girls giving birth before turning 18 years old by 2020 globally [1]. About 90% of the births occurred in developing countries with Sub-Saharan Africa [SSA] carrying the biggest burden at 19.3% by 2019 [2].

The outbreak of COVID-19 pandemic worsened the teenage girls’ vulnerability to early and unwanted pregnancies [3]. In Uganda, the rate of teenage pregnancy in some districts increased by 30%-50% during the COVID-19 pandemic period of 2021 compared to the pre-COVID-19 pandemic period [4–6]. The COVID-19 pandemic- related restrictions such as lockdown of people and closure of schools in particular affected the routine delivery of and access to the sexual reproductive health and contraceptive services including outreaches to schools where the majority of the in-school teenage girls could have easily been accessed with services [3]. Worse still, sexual and reproductive health SRH information is much harder to come by in crisis settings, leaving teenage girls unable to make informed reproductive and contraceptive choices [7]. Closure of schools and thus the free time during the out of school period increased the possibilities of the teenagers getting exposed to risky behaviors such as night clubs, alcohol consumption, and drug use and their associated risky sexual behaviors among others.

A number of countries with data have illustrated how the COVID-19’s outbreak exacerbated teenage girls’ vulnerability to early and unintended pregnancies and the consequences that come along as the number of cases continues to rise. In Kenya for example, 152,000 teenage girls became pregnant in the three months of COVID-19 lockdown, which was a 40% increase from 380,000 to 532,000 cases [8]. In Uganda, 354,736 teenage pregnancies occurred during the COVID-19 pandemic, which was a 55% increase from 354,736 to 551,235 teenage pregnancy cases occurring in the first six months of 2021 [9].

The rampant cases of teenage pregnancies during the COVID-19 pandemic occurred amidst the evidence that teenage girls in the age group of 13–19 are generally twice more likely to die from pregnancy and childbirth-related complications due to their low level of education, low level of antenatal care attendance, high risk of pregnancy induced hypertension, pre-eclampsia and eclampsia compared to the older women [10]. Worse still, evidence indicate that children born to teenage mothers are generally also 50% more likely to die in infancy than those born to women in their twenties or older due to premature labor and thus premature delivery with increased risk of neonatal morbidity [10].

The COVID-19 pandemic contributed to the limited delivery of the SRH services [6, 11], thus, limited availability and access to SRH services. The availability of contraceptive methods during crises like COVID-19 pandemic was limited due to programmatic shift from routine service delivery to the prevention and control of the pandemic [12]. Studies conducted in Uganda have documented the high prevalence of teen pregnancy during the COVID-19 pandemic period and attributed it to the harsh economic stress, anxiety as well as lack of access to contraceptive services and idleness [5, 6, 13].

The Ugandan government continues to be committed to increasing the use of modern contraceptive methods so that every sexually active Ugandan women of reproductive age can choose when and how many children she wants to have [1]. To reduce the risk of teenage pregnancy, several policies and adolescent pregnancy prevention programs are being implemented in Uganda including the setting of 18 years as the minimum age at which teenage girls can give informed consent to sexual activity, the national affirmative action on girl child education which mandates Universities and other tertiary institutions to add 1.5 points to every female student joining any University and other tertiary institutions for educational programs, and the creation of adolescent friendly reproductive health services within the health sector [14, 15]. However, the COVID-19 related lockdown halted the smooth delivery of the SRH services such as outreaches to schools [8, 11]. Arguably, the COVID-19 related lockdown and closure of schools availed the teenage girls with plenty of time to engage in sexual relations with men, which unfortunately carried the risk for frequent sexual activity, teenage marriage, sexual violence, and teenage pregnancy [8, 11, 12].

Hoima district located in western Uganda was among the districts with a high rate of teenage pregnancies among in-school teenage girls even before the COVID-19 pandemic. The prevalence of teenage pregnancy among in-school teenagers aged 15 to 19 years in Hoima district stood at 29% in 2019 [16] which was a year just before the commencement of the series of COVID-19-related restrictions and lockdown from March 2020 onwards. The 29% teenage pregnancy rate in Hoima district was higher than the national rate for adolescent pregnancy and childbearing of 25% in 2019 [17].

Despite the aforementioned high prevalence of teenage pregnancy in Uganda generally during the COVID-19 pandemic and Hoima district in western Uganda in particular even before the COVID-19 pandemic, previous research on the impact of the COVID-19 pandemic on the sexual and reproductive health of in-school teenage girls’ population in Uganda remained scarce. This study therefore determined the prevalence and factors associated with teenage pregnancies among in-school teenage girls during the COVID-19 pandemic.

Information on SRH issues among the school-going teenage girls during the COVID-19 pandemic would inform policies, and strategies for increasing delivery and access to SRH services among vulnerable populations in this case the school-going teenage girls during pandemics. The study findings may also inform the development of COVID-19 recovery and resilience strategies for SRH targeting teenage girls in SSA.

## Methods

### Study design and setting

We conducted a descriptive cross-sectional community-based study employing quantitative methods. The study was conducted in three sub-counties of Kyabigambire, Buhanika, and Buhimba, Hoima district. Hoima district is located in western Uganda, 230 kilometers from Kampala, Uganda’s capital city. The prevalence of teenage pregnancy in Hoima district among in-school teenagers aged 15 to 19 years was 29% in 2019 which was above the Uganda’s national average of 25% in 2019 [16].

### Study participants and sample size

The study population was in-school teenage girls aged 13 to 19 years who were residents of Hoima district in Western Uganda. The sample size was estimated using Kish & Leslie 1965 formula [18]. The sample size was calculated based on the considerations that Z = 1.96 at 95% confidence level, d = allowable error of 5%, p = 25% of teenage pregnancy and childbearing rate in Uganda [13]. The Uganda national average rate for teenage pregnancy of 25% was used for the sample size calculation instead of the 29% teenage pregnancy rate for Hoima district because the dataset used to calculate the 29% teenage pregnancy rate for Hoima district did not include the 13–14-year-old teenage girls which the current study included. After adjusting for a non-response rate of 10%, we obtained a sample size of 318 participants. Being a study on a sensitive and potentially stigmatizing research topic of sexual behaviors and teenage pregnancy which carries high chances of some participants declining to respond non-response to some of the key questions in the questionnaire, adjusting the sample size for non-response rate was critical for minimizing missing data for key outcome and independent variables of the study. The sample size was not adjusted for design effect because the pilot study indicated no intra-cluster correlation [ICC] with ρ = 0. The representativeness of the sample was assured through the use of the probability sampling method multi-stage sampling.

### Sampling method and participant recruitment procedures

The study participants were selected using a multi-stage sampling method. Three sub-counties out of 13 sub counties in Hoima district were randomly selected by random sampling. All of the sub-counties were written on separate pieces of paper, which were then randomly selected with replacement. Simple random sampling was used at the parishes within the three sub-county level to identify two parishes within each sub-county totaling 6 parishes. The names of each sub-county’s parishes were written on separate papers, folded and mixed thoroughly, and the researchers then chose two papers at random with replacement. Using simple random sampling, two villages were selected from each of the 6 parishes totaling 12 villages. The study enlisted the participation of 27 households from the 12 villages. The households were chosen using random route sampling. The local political leaders by village also known as local council I LCIs were used to locate the center of the village, and a pen was dropped to determine the direction of movement of the field research team to identify the first household. The LCIs also guided the field research team to the rest of the households with a teenage girl until the required sample size was attained.

### Data collection tool

Interviewer-administered questionnaire were used to collect the data. The items in the questionnaire were researcher-developed based on the relevant literature [19–21]. Risk and predisposing factors for teenage pregnancy were identified and adjusted for the local setting using the Uganda demographic health survey [17]. Literature indicated potential predictors of teenage pregnancy during the COVID-19 pandemic which included socio-demographic, individual, and environmental factors. The tool was pretested during a pilot study on 30 in-school teenage girls in Barapwo ward Lira district northern Uganda. Reliability index Cronbach alpha test of the tool yielded a reliability coefficient of r equals to 0.82 which indicated the tool had a good internal consistency. Adjustments were made to the items of the tool based on the pretest findings to improve the reliability and validity before its use for data collection in the actual study.

In the questionnaire, the socio-demographic factors considered were age, place of residence, marital status and religion. Individual factors considered were repeated teenage pregnancy, drug use, number of sexual partners, contraceptive use, sexual activity status, receipt of sexual education, experiences of gender-based violence, coercion, rape, forced marriage, peer pressure, idleness at home, perceptions regarding level of access to health facilities, SRH services, culture and its promotion of teenage pregnancy, access to SRH information, and distance from the nearest health facility. Environmental factors considered were policy-related factors, community-related factors, family-related factors, and health-system-related factors. The data collection tool was pretested during the pilot study and necessary adjustments were made to improve its reliability, face and content validity.

### Operational definitions and measures of key study variables

Teenage pregnancy was defined as conception or pregnancy or child birth by any teenage girl aged 13 to 19 years. And to measure the occurrence of teenage pregnancy during the COVID-19 pandemic period among the teenage girls in this study, participants regardless of their pregnancy, delivery nor child birth status were asked whether they have ever conceived or gotten pregnant from sexual intercourse or given birth during the COVID-19 pandemic period of January 2021 to December 2021 or not.

Forced marriage was defined as a marriage or union involving the teenager girl by force or by arrangements involving parents without the teenage girl’s informed assent or consent to the marriage. Therefore, to measure teenage forced marriage among the teenage girls in this study, we asked the participants who were found married whether their marriage at the teenage age was their free will or forced against their will or an arrangement involving their parents or guardians against their will or not.

Peer pressure refers to the direct or indirect influence of the teenage peers on a teenage girl’s sexual behavior. Therefore, peer pressure among the teenage girls in this study was measured by asking the participants whether their indulgence in the sexual intercourse during the COVID-19 pandemic period was as a result of an influence or pressure from their peers, attempt to behave like their peers or not.

Coercion refers to a teenage girl being persuaded to indulge into sexual activity using force, duress or threats of punishment. Therefore, to assess coercion among the teenage girls in this study, the participants were asked if their indulgence into sexual activity during the COVID-19 pandemic period was as a result of force, duress or threats of punishment from third-parties including their parents or guardians or not.

Sexual education refers to receipt of any form of advice or information or reading materials like posters or brochures or flyers or counseling or coaching or lectures or training or seminars or group discussions by the teenage girls on topics related to sexual risks, safer sexual behaviors, dangers of teenage pregnancy and motherhood, sexuality and reproductive health to enable them make safer sexual and reproductive choices. To assess receipt of sexual education among the teenage girls in this study, the participants were asked if they have ever received any form of advice or information or reading materials like posters or brochures or flyers or counseling or coaching or lectures or training or seminars or group discussions on topics related to sexual risks, safer sexual behaviors, dangers of teenage pregnancy and motherhood, sexuality and reproductive health to enable them make safer sexual and reproductive choices or not.

Current use of contraceptive methods was defined as current use of modern contraceptive methods including intentional use of modern contraceptive devices, drugs, chemicals, and hormones to prevent unintended or unwanted conception or pregnancy. Relatedly, current use of contraceptive method among the teenage girls in this study was assessed by asking the teenage girls whether they were currently using or ever used during the COVID-19 pandemic period any modern contraceptive method or devices or drugs or chemicals or hormones to prevent unintended or unwanted conception or pregnancy or not.

Access to SRH information refers to the perceived ability or limitations in getting information about SRH services from sources within their reach. Therefore, access to information among the teenage girls in this study was assessed by asking the participants whether they feel they were able to or limited in getting or obtaining information about SRH services from sources within their reach or not.

Sexual activity refers to any act of sexual intercourse. A teenage girl is regarded as sexually active if they engaged in sexual intercourse within 3 months prior to the study and sexually inactive if the sexual intercourse they engaged in happened at a period beyond 3 months prior to the study. Therefore, to assess the level of sexual activity among the teenage girls in this study, participants were asked whether they have engaged in sexual intercourse within the past 3 months or earlier than the past 3 months.

### Outcome variable

The primary outcome for this study was teenage pregnancy among teenage girls aged 13–19 years. Teenage pregnancy was measured by asking the participants regardless of their school type, marriage, pregnancy, delivery nor child birth status the questions “Are you pregnant? “, “Do you have a child?”, and ‘If yes, to any of the above questions, did you get pregnant during the pandemic period of January 2021 to December 2021 or not?’ The response options to all the aforementioned questions were either ‘Yes’ or ‘No’. The questions were then used to come up with a binary outcome variable that typify the occurrence of teenage pregnancy during the COVID-19 pandemic period of 2021.

To mitigate negative reactions to the personal nature of the items to measure the outcome variable teenage pregnancy status, private spaces such as nearby tree shades around the participant’s home without interference from a third-party was secured and used for the data collection sessions. More so, the Research Assistants established a good rapport with each of the participants prior to the data collection sessions. Also importantly, trained female Research Assistants with health background and field research experience were used to conduct the data collection sessions. For participants who were found to have given birth and had living children, the Research Assistants asked to take a look at the children. For participants who were found to still be pregnant, the apparent visible pregnancy status was noted against their responses to the item if they were pregnant.

To ensure accuracy of data, the participants were asked to state months or the gestation of the pregnancy if they were still pregnant, date of delivery or birth and the age of the children if they already gave birth. Participants were also asked to provide any medical record regarding the pregnancy, delivery or child birth such as pregnancy test results, antenatal care attendance cards, delivery and maternity discharge forms, or infant immunization cards and school report cards. The aforementioned confirmatory items and documentary data sources helped to ensure the accuracy of the participants’ age, teenage pregnancy status and whether or not it occurred in the COVID-19 pandemic period of January 2021 to December 2021.

### Data collection

Data was collected from December 2021 to January 2022. Three experienced Research Assistants fluent in both English and local language Runyooro were hired and trained on the data collection tool and procedures. The Research Assistants visited the selected villages and used the village leaders also known as LCIs to develop the sampling frames the total list of households with in-school teenage girls aged 13–19 years regardless of their pregnancy, delivery nor child birth status by village. The LCIs also field guided the Research Assistants to the sampled households with eligible girls for the data collection. Data collection process took place in private spaces at the participant’s home. Each interviewer-administered questionnaire took 10 to 20 minutes. Collected data were recorded with pen directly on the questionnaire by the Research Assistants.

### Data management and statistical analysis

The Epi Data version 3.1 software was used for double entry and cleaning of the data. The validated data were exported to STATA version 15.0 for statistical analysis. Descriptive statistics were used to summarize the participant’s socio-demographic data and also to analyze for the prevalence of teenage pregnancy which is the primary study outcome variable. We conducted multi-collinearity analysis using Variance Inflation Factor VIF for the two independent variables with numeral data i.e. the participant’s number of sexual partners and the distance of residence from the nearest health facility and found an VIF value of <10 which showed no correlation between the independent variables. For the rest of the independent variables whose data were categorical in nature, we conducted bivariate binary logistic regression for associations between them and the outcome variable. We selected the independent variables associated with the outcome variables at p-values ≤0.05 from bivariate analysis for entry into the Multivariate regression model. Multivariate binary logistic regression model was entered to identity significant independent predictors for teenage pregnancy. Crude odds ratios COR, adjusted odds ratios AOR and their 95% Confidence Interval CI were calculated for the measures of association. Associations with p-value of ≤0.05 were considered to be statistically significant.

### Ethical approval and consent to participate

Ethical approval for the study was obtained from the Gulu University Research Ethics Committee with the approval number GUREC-2021-114 because Lira University did not have its own accredited Research Ethics Committee at the time of the study. Prior participant screening was conducted to determine the eligibility of the potential participants to participate in the study. Informed consent was obtained from all the participants’ guardians or parents, as well as assent from the participants themselves. Pregnant teenage mothers were considered emancipated minors and gave informed consent. The purpose of the study, methods of data collection, study time frame, potential risk, benefits, and freedom to refuse or withdraw participation from the study without penalties were explained to the participants before enrollment into the study. Informed consent forms were obtained by the Research Assistants. Informed consents were obtained by written signature or thumb print. Participants were assured of confidentiality of the data. The study data collection tools were anonymous. During the actual data collection, privacy was assured to the teenage girls by ensuring they were interviewed in places that they felt comfortable to share information and away from their parents or guardians unless they said they were okay with their presence. Hard copy data were kept under lock and key only accessible to the research team. Electronic databases were password protected and the password was only known to the research team.

## Results

Of the 318 targeted samples, all the 314 teenage girls who were approached by the study Research Assistants to take part in the study provided informed consent and participated in the study giving a response rate of 98.7%. Four eligible girls could not be found in the selected villages within the study data collection timeframe.

The results in Table 1 show that the participant’s median age was 16 years and the age range was 13–19 years respectively. Of the 314 participants, 218(69.4%) were single, 178 (56.7%) were Protestants by religious affiliation and 165(52.5%) were secondary school students.

**Table 1:** Prevalence of pregnancy and its associated factors among the teenage girls aged 13–19 years in Hoima district western Uganda during the COVID-19 pandemic in 2021.

Variable	Pregnant N (%)(n = 96)	Not pregnant N(%) (n = 218)	Total N(%)	COR [95% CI]	P-value
**Teenage pregnancy**	96 (30.6%)	218 (59.1%)	318 (100)		
**Socio demographics**					
**Marital status**					<0.001**
Married	69(71.8)	27(28.2)	96 (30.6)	1.00	
Un married	27(12.4)	191(87.6)	218(69.4)	18.07(9.92–32.9)	
**Age group**					<0.000**
13–17 years	50(20.8)	190(79.2)	240(76.4)	1	
18–19 years	46(62.1)	28(37.9)	74(23.6)	0.16(0.09–0.28)	
**Religion**					0.052
Catholic	32(40.0)	48(60.0)	80(25.5)	1.0	
Protestant	44(24.7)	134(75.3)	178(56.7)	2.03(1.15–3.56)	
Muslim	89(29.6)	19(70.4)	27(8.6)	1.58(0.61–4.05)	
Others	12(41.4)	17(58.6)	29(9.2)	0.94(0.95–2.24)	
**Level of education**					0.036*
Primary	37(24.8)	112(75.2)	149(47.5)	1.0	
Secondary	59(35.8)	106(106)	165(52.5)	0.59(0.35–0.96)	
**Type of school**					0.046*
Day	71(34.3)	136(65.7)	207(65.9)	1.00	
Boarding	25(23.4)	82(76.6)	107(34.1)	1.71(1.00–2.91)	
**Individual factors**					
**Gender-Based Violence**					0.0018**
Yes	17(56.6)	13(43.4)	30(9.6)	1.0	
No	79(27.8)	205(72.2)	284(90.4)	3.393(1.57–7.3)	
**Coercion**					0.273
Yes	8(42.1)	11(57.9)	19(6.0)	1.0	
No	88(29.8)	207(70.2)	295(94.0)	1.71(0.66–4.39)	
**Rape**					0.834
Yes	4(33.3)	8(66.7)	12(3.8)	1.0	
No	92(23.3)	302(76.7)	302(96.2)	1.14(0.34–3.88)	
**Number of sexual partners**				0.37(0.25–0.54)	<0.001**
**Current use of contraceptive methods**					<0.001**
Yes	54(54.0)	46(46.0)	100(31.8)	1.00	
No	42(19.6)	172(80.4)	214(68.2)	4.8(2.86–8.07)	
**Sex for gifts or money**					<0.001**
Yes	66(57.4)	49(42.6)	115(36.6)	1.00	
No	30(15.1)	169(84.9)	199(63.4)	7.6(4.44–12.96)	
**Sexual activity**					<0.001**
Sexually Inactive	74(59.6)	52(40.4)	126(41)	1	
Sexually Active	22(11.8)	166(88.2)	188(59)	10.73(6.07–18.96)	
**Idle at home**					<0.001**
Yes	61(43.9)	78(56.1)	139(44.3)	1.0	
No	35(20.0)	140(80.0)	175(55.7)	3.13(1.89–5.15)	
**Limited access to health facilities**					<0.001**
Yes	24(32)	51(68.0)	75(23.9)	1.00	
No	72(30.1)	167(69.9)	239(76.1)	1.09(0.62–1.90)	
**Peer pressure**					0.10
Yes	38(40.9)	55(59.1)	93(29.6)	1.0	
No	58(26.2)	163(73.8)	221(70.4)	1.94(1.16–3.23)	
**Culture increases the risks of teenage pregnancy**					<0.001**
Yes	29(49.2)	30(50.8)	59(18.8)	1.0	
No	67(26.3)	188(73.7)	255(81.2)	2.71 (1.52–4.85)	
**Forced marriage**					<0.001**
Yes	23(59.0)	16(41.0)	39(12.4)	1.00	
No	73(26.5)	202(73.5)	275(87.6)	3.97(1.99–7.95)	
**Received sexuality education during COVID-19**					0.003*
Yes	54(54.0)	46(46.0)	173(55.1)	1.00	
No	42(19.6)	172(80.4)	141(44.9)	0.49(0.29–0.79)	
**Access to information**					0.217
Yes	72(31.3)	158(68.7)	230(73.2)	1.00	
No	24(28.6)	60(71.4)	84(26.8)	1.32(0.65–1.977)	
**Environmental factors**					
**Availability of health workers during Lockdown**					1.791
Available	89(31.8)	191(68.2)	280(89.2)	1.00	
Not available	7(20.6)	27(79.4)	34(10.8)	1.79(0.75–4.28)	
**Availability of Adolescent friendly services**					2.668
Available	90(32)	191(68.0)	172(54.8)	1.00	
Not available	6(18.2)	27(81.8)	142(45.2)	0.84–5.31	
**Fear to attend health facilities due to COVID-19**					0.10
Yes	53(30.8)	119(69.2)	281(89.5)	1	
No	43(30.3)	99(69.7)	33(10.5)	(0.63–1.66)	
**Distance from health facility**		0.901		0.95(0.87–1.03)	0.901

### Teenage pregnancy and associated factors among in-school teenage girls during the COVID-19 pandemic

The results in Table 1 indicate that 96 (30.6%) of the teenage girls have ever gotten teenage pregnancy during the COVID-19 pandemic. Teenage pregnancy was associated with the teenage girl’s marital status, age, level of education, and type of school, experiences of gender-based violence, number of sexual partners, contraceptive usage, indulgence in sex trade and sexual activities. Other factors associated with the occurrence of teenage pregnancy among the in-school teenage girls were being idle at home, access to health facilities, culture, forced marriage and sex education.

### Predictors of teenage pregnancy among in-school teenage pregnancy among in-school teenagers during the COVID-19 pandemic

After adjusting for other variables such as level of education, number of sexual partners, sexual activity, access to health facilities, forced marriage and others. In the model the variables that had higher odds of teenage pregnancy (Table 2) were being an unmarried teenager (aOR: 9.6; 95%CI: 4.64–19.87; p = 0.000), being in boarding school (aOR 2.83, 95%CI 1.36–5.86, p = 0.005), contraceptive use (aOR: 2.54; 95%CI: 1.12–5.4; p = 0.015) and being involved in sex trade (aOR 3.16, 95%CI 1.5–6.7, p = 0.003). However, those that had reduced odds of teenage pregnancy included: being and older teenager (aOR: 0.15; 95%CI: 0.07–0.32; p = 0.000) and not being sexually educated during the period (aOR 0.36, 95%CI 0.13–0.62, p = 0.024).

**Table 2:** Predictors of teenage pregnancy among in-school teenage girls aged 13–19 years in Hoima district western Uganda during the COVID-19 pandemic in 2021.

Variable	COR (95% CI)	AOR (95% CI)	P-value
**Marital status**			
Married	1.00	1.00	
Un married	18.07(9.92–32.95)	9.6(4.64–19.87)	**<0.001** **
**Age**			
13–17		1.00	
18–19	0.55(0.48–0.65)	0.15(0.07–0.32)	**<0.001** *
**Type of school**			
Day	1.00	1.00	
Boarding	1.71(1.00–2.91)	2.83(1.36–5.86)	**0.016** *
**Contraceptive use**			
Yes	1.00	1.00	
No	4.8(2.86–8.07)	2.54(1.12–5.4)	**0.006** *
**Sex for gifts or money**			
Yes	1.00	1.00	
No	7.6(4.44–12.96)	3.16(1.5–6.7)	**0.001** *
**Sexual education**			
Yes	1.00	1.00	
No	0.49(0.29–0.79)	0.44(0.23–0.88)	**0.006** *

*level of significance at p<0.05

**level of significance at p<0.001, p≤0.05

## Discussion

We found the prevalence of teenage pregnancy among in-school teenage girls in Hoima District Uganda during the COVID-19 pandemic of 2021 was 30.6% which was higher than the global, national, and western Uganda also known as Bunyoro region of Uganda prevalences for teenage pregnancy of 15%, 25%, and 29%, respectively before the COVID-19 [17, 22]. This finding tended to suggest that the COVID-19 pandemic contributed to a small increase in the prevalence of teenage pregnancy among the in-school teenage girls in Hoima district Uganda. The finding showed that teenage pregnancy is a public health problem in Uganda and the pandemic did not that much increase the teenage pregnancy in Uganda contrary to the previous assertions that the COVID-19 pandemic worsened the teenage girls’ vulnerability to early and unwanted pregnancies [3]. The slightly higher prevalence of teenage pregnancy during the COVID-19 pandemic could be that, during a crisis, the conditions that erode women’s ability to exercise bodily autonomy and reproductive choices increase catastrophically, hence increasing the risks of unintended pregnancy [7]. The unexpectedly slighter than drastic increase in the prevalence of teenage pregnancy during the COVID-19 pandemic period observed in Hoima district Uganda can arguably be attributed to the possibility that the COVID-19 related restrictions and lockdown measures provided a-stay-home environment for the otherwise in-school teenage girls to closely associate and learn from their paternal aunties who are by the Ugandan culture the sexual reproductive health counselors for teenage girls and young women. In Ugandan extended family settings, these paternal aunties are mandated to counsel and actually coach their nieces and other young girls in the area on SRH matters including how to minimize the risk of unintended pregnancies.

The study results from Hoima district Uganda when compared to the study conducted in Kenya which found that in-schools teenage girls were two times more likely to become pregnant during the COVID-19 pandemic compared to the pre-COVID-19 era [23] are in agreement in terms of the increase in the prevalence of teenage pregnancy during the COVID-19 pandemic compared to the pre-COVID-19 period but not that consistent in terms of the magnitude of the increase of the teenage pregnancy during the COVID-19 pandemic. As noted earlier, the observed slighter than drastic increase in the prevalence of teenage pregnancy among the teenage girls during the COVID-19 pandemic period in Hoima district Uganda compared to the previous study conducted in Kenya which showed drastic increase could possibly be due to the intervention of the Ugandan paternal aunties who are by the Ugandan culture the SRH counselors for teenage girls and young women which may not be the culture and practice in Kenya. The research findings suggests that multi-sectorial and multi-faceted approaches and programs be devised to satisfy the SRH needs of the in-school teenage girls in the presence or absence of an emergency crisis.

Our results indicated that unmarried teenage girls in Hoima district Uganda were more than 9 times more likely to get pregnant during the COVID-19 pandemic compared to their counterparts. This finding contradicts a number of previous studies that showed that being married increases the risk of teenage pregnancy [20, 24, 25]. This finding was unexpected but it’s understandable because during the COVID-19 pandemic, unmarried teenage girls were idle at home and could have misused their free time in unintended pregnancy-risky behaviors such as visiting night clubs, alcohol and drug use compared to how they would be occupied with studies at schools [23]. The results imply that the SRH needs of unmarried teenage girls should be given attention during emergencies to prevent unintended pregnancies.

Teenage girls who never used modern contraceptive methods were found to be almost 3 times more likely to become pregnant during the COVID-19 pandemic period compared to their contraceptive-using counterparts. This finding was consistent with a number of previous studies conducted in Uganda and other SSA countries [26–28]. It is the fact that, when used correctly, modern contraceptive methods are effective in preventing pregnancy among sexually active teenage girls. It was a possibility that the COVID-19 pandemic related restrictions and lockdown measures particularly the restrictions on the use of public transport affected the teenage girl’s access to the sources or providers of the contraceptive methods, and consequently increasing their risk to teenage pregnancies [29]. The above finding implies that special delivery mechanisms for modern contraceptive methods be devised for vulnerable populations like teenage girls alongside pandemic related restrictions and lockdown rather than relying on the routine statistic health facilities as the sole source of modern contraceptive methods for all categories of women particularly the teenage girls.

Adult teenage girls (those aged 18–19 years) had a lower risk of becoming pregnant during the COVID-19 pandemic period compared to the juvenile teenage girls (those aged below 18 years). This finding contradicts the findings of Ayanaw and colleagues, who found that adult teenage girls were more likely to become pregnant compared to the juvenile teenage girls [30]. This discrepancy could be explained by the differences in study designs whereby the previous study by Ayanaw et al were conducted among the teenage girls in the age group of 15 to 19 years excluding the 13–14 year-olds based on the assumption that they were more sexually active than those aged 13 to 14 years who were included in our study in Hoima district Uganda. With the COVID-19 restrictions, juvenile teenage girls may not have received the attention they deserved in terms of SRH information, education, communication and services, and thus more likely to engage in unsafe sexual activity than their adult counterparts. Agreeable, the data from Hoima district Uganda and other previous data all suggest that closure of schools and thus keeping of young girls out of schools can have a significant influence on their sexual and reproductive health outcomes [31, 32].

Our study findings from Hoima district Uganda showed that studying from a boarding school was associated with an increased likelihood of getting a teenage pregnancy during the COVID-19 pandemic compared to studying from a day school. This finding is consistent with the finding of a previous study conducted in the same area by Kasozi and colleagues who found that girls in the school’s boarding section were more likely to become pregnant when they return home for holidays compared to their counterparts in the school’s day section [16]. It is possible that being in day school provides unique opportunities for the teenage girls to acquire life skills including awareness of sources of SRH services, safer sex negotiation and self-defense skills which exposures are often lacking for the girls in the boarding section of Ugandan schools. The finding calls for the integration of life skills training including sex education into the intra and extra curricula activities of boarding schools in Uganda.

During the COVID-19 pandemic, having not received sex education was associated with a lower risk of teenage pregnancy. This finding contradicts previous studies that showed that sex education also known as sexuality education lowers the risk of teenage pregnancy [33, 34]. The difference in the study findings could be due to the differences in the cultural beliefs and practices regarding sexuality in the study settings. In the case of current study setting in Hoima district Uganda, sex education was not yet by policy being conducted in schools [35]. Even at the household level, teenage girls in Uganda are most likely to be educated about SRH issues when it is too late or at the time when the teenage girls are already pregnant due to the traditional cultural beliefs and taboos that forbid sexual related communication between adults especially parents and their young children including teenage girls. Therefore, in our study setting in Hoima district Uganda, it was highly likely that the teenage girls who reported to have received sex education could have received the education from their sexual partners during unsafe sexual activities and thus the increased risk of teenage pregnancy observed in this study. The aforementioned study finding calls for a national policy on sex education and formalized school and community-based sex education strategies for teenage girls and boys in Uganda.

The study in Hoima district Uganda found a higher risk of teenage pregnancies among teenage girls who engage in sex trade for material gifts or money compared to their counterparts. In fact, teenage girls in Hoima district Uganda who had ever engaged in the sex trade for material gifts or money were almost 4 times more likely to report teenage pregnancy compared to their counterparts. It is reasonable that teenage girls who engage in the sex trade for gift or money may lack the ability to negotiate for safer sex for fears of losing the gift or money which unfortunately put them at a higher risk of unwanted teenage pregnancy and sexually transmitted diseases. This study finding from Hoima district Uganda is in line with a study by Barnert and colleagues (2020) which found that commercially sex involved girls were more likely to get unwanted pregnancy compared to their counterparts [36]. The rampant sex trade among teenage girls (standing at 36.6%) coupled with high prevalence of unwanted teenage pregnancy found in western Uganda during the COVID-19 pandemic calls for national policies on adolescent friendly community distribution channels for antiretroviral drugs (ARVs) for pre-exposure prophylaxis (PrEP) and or post-exposure prophylaxis (PEP) as HIV prevention biomedical methods for high-risk populations such as teenage girls who engage in commercial sex.

The current study collected data from adequate sample of in-school teenage girls of a wide age range of 13–19 years and irrespective of their school type, marriage, pregnancy, delivery or child birth status. The study also helped to provide clarifications on the situation of teenage pregnancy during the COVID-19 pandemic in terms of the prevalence, the associated factors and the predictors in Hoima district Uganda. Nonetheless, the teenage pregnancy status was self-reported by the study participants and thus the method carried the risk of underreporting as some of the participants may not be aware of their pregnancy status at the time of the study. Our study in Hoima district Uganda was based on the previous research findings that indicated an increased rate of teenage pregnancy during the COVID-19 pandemic period compared to the pre-COVID-19 pandemic period, and thus data weren’t collected to ascertain whether the pre-COVID-19 prevalence of teenage pregnancies was significantly different from the prevalence during the COVID-19 pandemic period among the study participants. More so, the use of the interviewer-administered questionnaire method carried the risk of social desirability bias with the potential of making some of the participants to answer some of the questions in a manner they viewed favorable to the interviewers. Key informant interviews of health workers, teachers, parents, and social workers could have provided alternative perspectives on the factors that contributed to some of the cases of teenage pregnancy during the COVID-19 pandemic period compared to the pre-COVID-19 pandemic period.

## Conclusion

Our study found that 3 out of 10 in-school teenage girls in Hoima district Uganda have ever gotten teenage pregnancy during the COVID-19 pandemic period of 2021. Teenage pregnancy was prevalent among teenage girls who were juvenile, not married, studying from boarding schools, never-used modern contraceptive methods and those engaged in sex trade. Teenage pregnancy was less prevalent among adult teenage girls and also less prevalent among teenage girls who did not receive sex education. The study findings from Hoima district Uganda calls for policies, contingency plans and programs aimed at delaying age for sexual activities, increasing modern contraceptive use and minimizing pregnancy risk from sex trade among the in-school teenage girls during COVID-19 pandemics.

## Supporting information

S1 File (RTF)

## List of abbreviations

COVID_19: Corona Virus Disease 2019
GUREC: Gulu university Research Ethics Committee
SRHR: Sexual reproductive health and rights
UBOS: Uganda bureau of statistics
UDHS: Uganda demographic health and survey
YFS: Youth friendly services
