# Supplementary material for: Prevalence and correlates of teenage pregnancy among in-school teenagers during the COVID-19 pandemic in Hoima district western Uganda–A cross sectional community-based study
Source: PLoS One. 2022 Dec 16;17(12):e0278772. doi: 10.1371/journal.pone.0278772 (PMC9757589; doi:10.1371/journal.pone.0278772)
Supplement: S1 File — (RTF) [file pone.0278772.s001.rtf]

Prevalence and correlates of teenage pregnancy among in-school teenagers during the COVID-19 pandemic in Hoima district Western Uganda – A cross sectional community-based study 
	

	CODE 	Questionnaire number	
	
	A	SECTION A: SOCIO-DEMOGRATIC CHARACTERISTICS  	
1	A1	What is your Age? 	…………………………	
2	A2	What is your marital status?	1.	Married
2.	Cohabiting 
3.	Single	
3	A2	What is your religion?	1.	Catholic
2.	Protestant
3.	Muslim
4.	Others 	
4	A3	What is your level of education?	1.	Primary 
2.	Secondary	
5	A4	Which type of school are you in?	1.	Day
2.	Boarding 	
6	A5	How do you describe your place of residence?	1.	Rural 
2.	Urban 	
	B	SECTION B: PREGNANCY STATUS 	
7	B1	Do you have a child? 
Show the health record? 	1.	Yes
2.	No	
8	B2	If yes, how old is the child in months? Any health record?	…………………….	
9	B3	If you have no child, are you pregnant? 
Show the health record?	1.	Yes
2.	No	
10	B4	If yes in B3 How old is the pregnancy (months)? Record?	…………………….	
	C	SECTION C: POTENTIAL FACTORS THAT MAYBE ASSOCIATED WITH TEENAGE PREGNANCY	
		PART A: INDIVIDUAL LEVEL FACTORS 	
11	C5	How many pregnancies have you had before?	……………………	
12	C6	Did you use drugs when school broke off (Alcohol, marijuana, shisha etc)?	1.	Yes
2.	No	
13	C7	Have you experienced any form of abuse after school broke off?
Which ones?	1.	GBV
2.	Child labor
3.	Coercion (threatened or duressed to have sex)
4.	Rape  
5.	Others specify ……………
6.	No I haven't experienced 	
14	C8	Are you currently using any contraceptive method?	1.	Yes
2.	No 	
15	C9	If yes which method is that? 	1.	Pill
2.	IUD
3.	Injection
4.	Implants
5.	Male condoms
6.	Female condoms
7.	Diaphragm
8.	Foam	
16	C10	When did you last have a sexual encounter?	1.	This week 
2.	This month 
3.	In the last six months
4.	Beyond six months 
5.	Never 	
17	C11	How many sexual partners do you have?	…………………………………	
18	C12	Have you ever had sex in exchange of anything (gifts and money)?	1.	Yes
2.	No 	
		PART B: POLICY LEVEL FACTORS 	
19	C13	Did closure of schools keep you idle at home? 	1.	Yes
2.	No	
20	C14	Did you get any sexual encounter after closure of schools?	1.	Yes
2.	No	
21	C15	Did lockdown limit access to hospitals for SRHR services (access to contraceptives)?	1.	Yes
2.	No	
		PART C: COMMUNITY LEVEL FACTORS 	
22	C16	Do your peers influence your sexual life while at home?	1.	Yes
2.	No	
23	C17	Do you know of any friends that got pregnant of recent (during COVID-19)?	1.	Yes
2.	No	
24	C18	Are girls stigmatized due to pregnancy in your community? 	1.	Yes
2.	No	
25	C19	Did some of your cultural practices put you at risk of getting pregnant while you are at home?	1.	Yes
2.	No	
		PART D: FAMILY LEVEL FACTORS 	
26	C20	Did your family force you to get married after closure of schools?	1.	Yes
2.	No	
27	C21	Did your family members offer sexuality education while at home? 	1.	Yes
2.	No	
28	C22	Does your family give more attention to boys to attend school?	1.	Yes
2.	No	
		PART E: HEALTH SYSTEM LEVEL FACTORS  	
29	C23	 Do you get information about sexual and reproductive services during COVID-19 pandemic?	1.	Yes
2.	No	
30	C24	Where do you usually get contraceptives during the school closure period?	1.	Pharmacy
2.	Public Health facilities 
3.	Clinics 
4.	Private hospitals 
5.	Others specify…………	
31	C25	Were OR Are health workers always at the health facilities to offer the services? 	1.	Yes
2.	No	
32	C26	Are the services friendly? 	1.	Yes
2.	No	
33	C27	Did you at any time fear to go to the health facilities because of COVID-19?	1.	Yes
2.	No	
34	C28	What is the distance to the nearest health center?	………………..................	
Any other information that you think would be helpful in this study?
………………………………………………………………………………………………………………………………………………
Thank you for your time and participation…. Have a wonderful day! 
